# Retraction Note: Type 1 diabetes and the challenges of emotional support in crisis situations: results from a randomized clinical trial of a multidisciplinary teleintervention

**DOI:** 10.1038/s41598-022-08539-9

**Published:** 2022-03-11

**Authors:** Janine Alessi, Alice Scalzilli Becker, Bibiana Amaral, Giovana Berger de Oliveira, Debora Wilke Franco, Carolina Padilla Knijnik, Gabriel Luiz Kobe, Ariane de Brito, Taíse Rosa de Carvalho, Guilherme Heiden Telo, Beatriz D. Schaan, Gabriela Heiden Telo

**Affiliations:** 1 Medical Science Program: Endocrinology, Universidade Federal do Rio Grande do Sul, Rua Ramiro Barcelos, 2350, prédio 12, 4° andar, Porto Alegre, RS 90035-003 Brazil; 2 Internal Medicine Department, Hospital São Lucas-Pontifícia Universidade Católica do Rio Grande do Sul, Porto Alegre, Brazil; 3 School of Medicine, Pontifícia Universidade Católica do Rio Grande do Sul, Porto Alegre, Brazil; 4 Medical and Health Sciences Program, Pontifícia Universidade Católica do Rio Grande do Sul, Porto Alegre, Brazil; 5 School of Medicine, Universidade Federal do Rio Grande do Sul, Porto Alegre, Brazil; 6 Endocrinology Division, Hospital de Clínicas de Porto Alegre, Porto Alegre, Brazil; 7 National Institute of Science and Technology for Health Technology Assessment (IATS)-CNPq/Brazil, Porto Alegre, Brazil

Retraction of: *Scientific Reports* 10.1038/s41598-022-07005-w, published online 23 February 2022

The Editors are retracting this Article.

Owing to an administration error this Article was inadvertently published before the peer review process was complete. The Editors apologise to the Authors and to readers for this error.

Author Janine Alessi stated on behalf of all co-authors that they agree with this retraction and its wording.

